# Comparison of the Quality of Hospitals That Admit Medicare Advantage Patients vs Traditional Medicare Patients

**DOI:** 10.1001/jamanetworkopen.2019.19310

**Published:** 2020-01-15

**Authors:** David J. Meyers, Amal N. Trivedi, Vincent Mor, Momotazur Rahman

**Affiliations:** 1 Department of Health Services, Policy, and Practice, Brown University School of Public Health, Providence, Rhode Island; 2 Providence VA Medical Center, Providence, Rhode Island

## Abstract

**Importance:**

In the Medicare Advantage (MA) program, Medicare enrollees may be steered by their health plan to specific hospitals. Little is known about the quality of hospitals that serve MA enrollees.

**Objective:**

To compare the quality of hospitals that admit MA enrollees with the quality of those that admit traditional Medicare enrollees.

**Design, Setting, and Participants:**

This cross-sectional study used data from the 2012 to 2016 Medicare Provider Analysis and Review to compare quality of care, as measured by the star rating given by the Centers for Medicare and Medicaid Services and readmission rates, in hospitals that serve MA enrollees and traditional Medicare enrollees using multinomial logit models. Participants were 7 130 610 Medicare beneficiaries admitted to 2994 acute care hospitals across the United States in 2016. Data were analyzed between August 2018 and August 2019.

**Exposures:**

The exposure was MA enrollment. Adjusters included demographic and clinical characteristics and zip code fixed effects.

**Main Outcomes and Measures:**

Hospital Compare star ratings and quintiles of performance in 30-day readmission rates.

**Results:**

The sample included 7 130 610 Medicare beneficiaries in 2016 (54.3% female; mean [SD] age, 72.7 [13.2] years). Of 12 190 270 total hospitalizations, 1 211 293 traditional Medicare and 494 352 MA patients were admitted to 718 low-readmission hospitals and 1 205 586 traditional Medicare and 526 955 MA patients were admitted to 597 high-readmission hospitals. Accounting for observed patient characteristics, MA enrollees less often entered either low- or high-quality hospitals and were more often admitted to average-quality hospitals. For nonemergent hospitalizations, MA enrollees were 1.9 percentage points (95% CI, 1.5-2.2 percentage points) less likely to enter a low-readmissions hospital, 5.1 percentage points (95% CI, 4.6-5.6 percentage points) more likely to enter an average-readmissions hospital, and 3.2 percentage points (95% CI, 2.9-3.5 percentage points) less likely to enter a high-readmissions hospital compared with traditional Medicare enrollees. Patients with MA were also 2.6 percentage points (95% CI, 2.2-2.9 percentage points) less likely to enter a 1- to 2-star hospital, 5.5 percentage points (95% CI, 4.9-5.9 percentage points) more likely to enter a 3-star hospital, and 2.8 percentage points (95% CI, 2.5-3.2 percentage points) less likely to enter a 4- to 5-star hospital compared with traditional Medicare enrollees. The differences were less pronounced for emergency admissions.

**Conclusions and Relevance:**

This study found that enrollees in MA plans were more likely to be admitted to average-quality hospitals instead of either high- or low-quality hospitals, suggesting that MA plans may be steering their enrollees to specific hospitals.

## Key Points

**Question:**

How does the quality of hospitals that admit Medicare Advantage enrollees compare with the quality of those that admit traditional Medicare enrollees?

**Findings:**

In this cross-sectional study of 12 190 280 hospitalizations in the United States in 2016, Medicare Advantage enrollees had a 2.8–percentage point lower probability of entering a highly rated hospital, a 5.5–percentage point higher probability of entering a hospital with an average ranking, and a 2.6–percentage point lower probability of entering a poor-quality hospital compared with traditional Medicare enrollees. Results were consistent across measures of hospital quality.

**Meaning:**

These findings suggest that, owing either to differences in preference or steering by Medicare Advantage contracts, Medicare Advantage enrollees are less likely to enter the highest- or the lowest-quality hospitals.

## Teaser

This cross-sectional study compares the quality of hospitals that admit patients enrolled in Medicare Advantage vs hospitals that admit patients enrolled in traditional Medicare.

## Introduction

More than one-third of all Medicare beneficiaries are now enrolled in the Medicare Advantage (MA)^1^ program, in which private insurance companies receive capitated payments to finance enrollees’ care needs.^2^ While MA plans are required to cover at least the same set of health care services as are covered by traditional Medicare (TM), MA plans can establish a preferred network of hospitals and health care practitioners for their enrollees. Very little is known about the differences in the quality of hospitals that serve MA enrollees compared with those serving TM enrollees.

In MA, the capitated payments that plans receive provide dual incentives to both reduce spending on unnecessary care and improve quality to prevent future expenditures. These incentives, and the flexibility MA plans have to steer their enrollees to specific practitioners, may lead to differences in the quality of care received by MA and TM enrollees. While a previous study^3^ found that MA enrollees are generally admitted to lower-quality nursing homes than TM enrollees, such differences in care quality cannot be generalized because MA enrollees historically use nursing home care at much lower rates compared with TM enrollees.^4^ Additionally, MA plans may emphasize primary care or hospital care that may reduce dependence on long-term care. Owing to a general lack of data on MA enrollees, it is not currently known what other differences in quality might exist.

Patient access to hospitals has recently come under increased scrutiny in the press^5^ and the academic literature.^6,7,8^ Little is known about how MA enrollees use hospitals. Recent studies^9,10^ have found that the size of MA plans’ hospital networks varies widely, with approximately 23% of plans having broad hospital networks and 16% of plans having narrow or ultranarrow networks. Furthermore, MA plans may limit some types of specialty hospital access. Medicare Advantage plans have been found to pay 5.6% less for hospital services than TM,^11^ which may also influence the hospitals available to MA enrollees. Several other factors can result in differences in the quality of hospitals to which MA and TM enrollees are admitted. Medicare Advantage enrollees and TM enrollees may have different geographic access to hospitals depending on where they live. Furthermore, MA and TM enrollees are known to differ in important ways^12^ and may have different preferences when selecting which hospitals they use for their care needs.

In this cross-sectional study, we compare the quality of hospitals to which MA and TM enrollees are admitted, accounting for enrollees’ characteristics and their geographic access to hospitals.

## Methods

### Data and Measures

This study was approved by the Brown University institutional review board and received a waiver of informed consent owing to the inability to contact enrollees in deidentified claims data. Reporting followed the Strengthening the Reporting of Observational Studies in Epidemiology (STROBE) reporting guideline. Study data were analyzed between August 2018 and August 2019.

Our primary data source for this analysis was 100% Medicare Provider and Analysis Review (MedPAR) claims, which include hospitalization-level data on all MA and TM enrollees who were admitted to a MedPAR reporting hospital.^13^ We used MedPAR and enrollment data from 2012 to 2016 to assess trends in hospital entrance over time. We then focused on data from 2016 for the remainder of our analysis. Since 2008, MedPAR has included claims from MA enrollees who were admitted to hospitals that receive Medicaid Disproportionate Share Hospital payments or graduate medical education hospitals, which accounts for 90% of all MA hospitalizations annually.^14,15^ From our initial sample of 3234 acute care hospitals, we removed 240 hospitals that do not receive Medicaid Disproportionate Share Hospital payments or medical education credits determined by hospital cost reports, as these hospitals are not required to report completely for MA enrollees, for a final sample of 2994 acute care hospitals, which account for more than 95% of all hospital records in the 2016 MedPAR data set.

Among 13 560 748 hospitalizations in 2016, we excluded 851 640 hospitalizations (6.2%) that took place out of an enrollees’ home state, as MA policies may differ for hospital access when an enrollee is traveling. We further excluded 518 838 hospitalizations (3.8%) that took place at non-MedPAR reporting hospitals, for a final sample of 12 190 270 hospitalizations among 7 130 610 Medicare enrollees. We included all Medicare enrollees (both those aged <65 years and those aged ≥65 years) in our study sample. In sensitivity analyses, we further excluded enrollees with a hospitalization in the prior 6 months, and only kept the first hospitalization of the year for each enrollee to check whether past hospitalizations are associated with future hospital choice. We stratified all hospitalizations by those that were admitted from the emergency department (8 608 120 hospitalizations) and those that were not (3 582 150 hospitalizations).

We used the Medicare Master Beneficiary Summary File to classify MA enrollees as those who were enrolled in the program for each month of 2012 to 2016 and assigned their MA status at the month they were admitted to the hospital. As of 2016, the Master Beneficiary Summary File includes monthly contract identification numbers for each enrollee that can be linked to publicly available MA star ratings and plan characteristics. For prior years, we linked enrollees to Healthcare Effectiveness Data and Information Set files, which have each enrollee’s contract number. For sensitivity analyses, we classified plans rated 4 or 5 stars as high quality and plans rated less than 4 stars as low to average quality to test whether there were further differences between different types of MA plans.

We linked each hospital admission to publicly available Centers for Medicare and Medicaid Services (CMS) 5-star hospital ratings for 2016, CMS adjusted 30-day readmission rates, and CMS adjusted 30-day mortality rates for acute myocardial infarction, stroke, heart failure, coronary artery bypass graft, and chronic obstructive pulmonary disease to classify admitted hospitals by quality. Our primary outcome of interest was whether an enrollee is admitted to a hospital in the lowest quintile of readmissions (low-readmissions hospitals), the second to fourth quintiles of readmissions (average-readmissions hospitals), or the highest quintile of readmissions (high-readmissions hospitals). We also assessed whether enrollees are admitted to low–, medium–, or high–star rated hospitals and to hospitals with different quintiles of 30-day mortality.

For enrollees in MA, we linked each enrollee’s plan and contract to publicly reported CMS MA characteristic files that provide details on the types of plans, plan premiums, plan MA star ratings, and the parent companies of plans.

### Statistical Analysis

Our primary analysis is from 2016 when the star ratings were released, and we stratified all our analysis by emergency and nonemergency admissions. We present the unadjusted and adjusted percentages of MA and TM enrollees who were admitted to different hospital quality categories in 2016. In sensitivity analysis, we also plotted admission trends from 2012 to 2016 to determine whether trends in admission changed over time.

To adjust for the observable patient characteristics that may be associated with the quality of the hospital beneficiaries enter, we fit separate multinomial logit models for admission to low-, average-, and high-readmissions hospitals. We also modeled entrance into hospitals of low (1-2 stars), average (3 stars), or high (4-5 stars) star rating quality and quintiles of 30-day mortality for several conditions. As geography is likely an important factor in determining the quality of hospitals available to beneficiaries, we used a Mundlak hybrid model to account for geographic differences.^16,17^ In the Mundlak model, we estimated multinomial logit regressions and included zip code–level means of all model covariates and clustered standard errors on the zip codes to approximate fixed effects. In secondary analyses, we included in eTable 2 in the Supplement results using only standard multinomial logit models as well as linear probability models estimating each outcome separately using zip code fixed effects.

In each model, we adjusted for patient age, sex, race/ethnicity, dual enrollment status, and a flag indicating whether patients were enrolled in MA or TM at the time of admission. We also included the distance each enrollee lived from the nearest high-, average-, or low-quality hospital. We stratified all our models by emergency and nonemergency admissions.

In sensitivity analyses, we further included covariates for intensive care unit use during the hospitalization and the Elixhauser Comorbidity Index score from the index hospitalization to evaluate whether patient acuity was associated with hospital selection (we cannot assess this fully, however, as both intensive care unit use and Elixhauser Comorbidity Index score assignment take place after hospital admission). We also stratified our results by less than 4 star– vs 4 or more star–quality MA plans, dual eligibility status, rural vs nonrural status, comorbidity status, and for the top principal diagnosis codes to determine whether the results are robust to different specifications. In additional specifications, we used inverse probability of treatment weights to balance observable differences in patient characteristics between MA and TM. We also estimated the models using multinomial logit models. The multinomial logit model is useful in that we could simultaneously assess the differences in the probability of entering a hospital of each rating category. The limitation, however, is that we were unable to account for the local neighborhood characteristics and availability of hospitals, as it is not appropriate to include fixed effects in nonlinear models.^18,19^

After assessing trends in admission between TM and MA enrollees, we conducted follow-up analyses on the MA population alone to evaluate what plan characteristics might be associated with the quality of admitting hospitals. We fit linear probability models adjusting for zip code fixed effects for each of the hospital quality outcomes described, including variables for MA plan contract star rating, whether the contract’s parent company is a national organization, tertiles of plan size, contract age, contract penetration in the enrollee’s county plan type (health maintenance organization [HMO], preferred provider organization, and other), and tertiles of plan premium. We clustered our standard errors in these models by the MA contract to account for multiple admissions from enrollees in the same contracts. All analysis was conducted using Stata software version 15 (StataCorp) and used 2-tailed significance tests with an α of .05.

## Results

The sample included 7 130 610 Medicare beneficiaries in 2016 (54.3% female; mean [SD] age, 72.7 [13.2] years). There were a total of 12 190 270 hospitalizations in 2994 acute care hospitals. We found 1 211 293 TM and 494 352 MA patients were admitted to 718 low-readmission hospitals, 1 159 142 TM and 522 258 MA patients were admitted to 1679 average-admission hospitals, and 1 205 586 TM and 526 955 MA patients were admitted to 597 high-readmission hospitals. Table 1 presents patient characteristics stratified by enrollment category and type of admission. Compared with TM enrollees, MA enrollees tended to be older (mean [SD] age for nonemergency admission, 70.6 [12.0] years vs 72.2 [10.3] years, respectively), were less likely to be white (for nonemergency admissions, 84.4% white vs 80.5% white, respectively), were less often dually enrolled in Medicaid (for nonemergency admissions, 17.6% vs 15.3%, respectively), and generally lived closer to higher-quality star-rated hospitals (for nonemergency admissions, median [interquartile range] distance to high-quality hospital, 5.4 [1.7-11.4] miles vs 4.4 [1.7-9.5] miles, respectively). The 3 most common nonemergency primary diagnoses were knee osteoarthritis, hip osteoarthritis, and coronary atherosclerosis. For the joint osteoarthritis diagnoses, hospitalizations were generally for joint replacement. The 3 most common emergency diagnoses were septicemia, pneumonia, and acute myocardial infarction.

**Table 1.** Characteristics of the Patient Sample

Characteristic	Nonemergency		Emergency	
	TM	MA	TM	MA
Patients, No. (%)	1 501 873 (69.0)	674 474 (31.0)	3 557 635 (71.8)	1 396 628 (28.2)
Age, mean (SD), y	70.6 (12.0)	72.2 (10.3)	74.2 (13.6)	74.6 (11.7)
Race, No. (%)				
White	1 274 043 (84.4)	542 715 (80.5)	2 865 001 (80.5)	1 062 103 (76.1)
Black	135 532 (9.0)	76 730 (11.4)	455 997 (12.8)	215 527 (15.4)
Other	38 700 (2.6)	20 751 (3.1)	78 528 (2.2)	34 232 (2.5)
Asian	14 694 (1.0)	11 949 (1.8)	56 341 (1.6)	23 571 (1.7)
Hispanic	24 242 (1.6)	20 652 (3.1)	83 172 (2.3)	57 927 (4.2)
Native American/American Indian	14 662 (1.0)	1677 (0.3)	18 596 (0.5)	3268 (0.2)
Female, No. (%)	809 615 (53.9)	372 285 (55.2)	1 966 286 (55.3)	778 615 (55.8)
Dual enrollment, No. (%)	264 813 (17.6)	102 906 (15.3)	950 854 (26.7)	324 972 (23.3)
Elixhauser Comorbidity Index score, mean (SD)	3.1 (2.0)	2.2 (2.1)	4.1 (2.1)	4.1 (2.1)
Any ICU use, No. (%)	327 275 (21.8)	128 122(19.0)	1 070 524 (30.1)	449 291 (32.2)
Top diagnosis codes, No. (%)^a^				
Knee osteoarthrosis	163 436 (10.9)	73 207 (10.9)	NA	NA
Hip osteoarthrosis	99 072 (6.6)	43 745 (6.5)	NA	NA
Coronary atherosclerosis	19 923 (1.3)	10 711 (1.6)	NA	NA
Septicemia	NA	NA	254 179 (7.1)	93 285 (6.7)
Pneumonia	NA	NA	122 361 (3.4)	44 960 (3.2)
Acute myocardial infarction	NA	NA	99 866 (2.8)	44 441 (3.2)
Distance to nearest hospital with high star rating, median (IQR), miles^b^	5.4 (1.7-11.4)	4.4 (1.7-9.5)	4.7 (1.7-10.4)	4.1 (1.6-8.8)

Abbreviations: IQR, interquartile range; MA, Medicare Advantage; NA, not applicable; TM, traditional Medicare.

^a^ Top diagnoses are defined by principal diagnosis codes on Medicare Provider Analysis and Review claims.

^b^ The distance to the nearest high rated hospital is calculated as the Euclidian distance from patient zip code centroid to hospital latitude and longitude.

To understand the role of neighborhood as a driver of disparity in the quality of hospital chosen, in the Figure, we plotted the percentage of TM and MA patients entering a hospital rated less than 3 stars, a hospital rated 3 stars, or a hospital rated 4 or more stars for nonemergent admissions by the distance from their residential zip code centroid to the nearest hospital of that rating. As expected, for all categories, patients who lived closer to a hospital with a given rating category were more likely to be admitted to a hospital of that rating; however, MA enrollees were less often admitted to hospitals rated less than 3 stars, more often admitted to 3 star hospitals, and less often admitted to hospitals rated 4 or more stars than TM enrollees who resided in the same zip codes. The same trend existed for emergency admissions (eFigure 2 in the Supplement); however, the magnitude of the differences was smaller.

**Figure.**
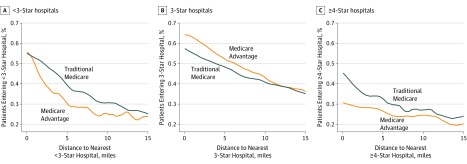
Share of Medicare Advantage and Traditional Medicare Patients Admitted to Hospitals in Neighborhoods With Different Proximity to Hospitals The graphs include nonemergency admissions only. Lines display the percentage of people who live a given distance from a given star-rated hospital who were admitted to that star quality hospital. If there were no selection due to Medicare Advantage enrollment status, these lines would be expected to overlap. Confidence intervals are not included; however, they are tightly aligned with the estimate lines and do not overlap.

In Table 2, we present the unadjusted percentages of patients in either TM or MA who were admitted to hospitals in each quality rating, readmission, and mortality category. We then present the adjusted difference based on the primary fixed-effects models. After adjusting for patient characteristics and accounting for geographic access, we found that MA enrollees were 1.9 percentage points (95% CI, 1.5-2.2 percentage points) less likely to be admitted to a hospital in the lowest quintile of readmissions, 5.1 percentage points (95% CI, 4.6-5.6 percentage points) more likely to be admitted to a hospital in the second to fourth quintiles of readmissions, and 3.2 percentage points (95% CI, 2.9-3.5 percentage points) less likely to be admitted to a hospital in the highest quintile of readmissions compared with TM enrollees. Medicare Advantage enrollees were also 2.6 percentage points (95% CI, 2.2-2.9 percentage points) less likely to be admitted to a 1- to 2-star hospital, 5.5 percentage points (95% CI, 4.9-5.9 percentage points) more likely to be admitted to a 3-star hospital, and 2.8 percentage points (95% CI, 2.5-3.2 percentage points) less likely to be admitted to a 4- to 5-star hospital compared with TM enrollees. Differences were similar, although substantially smaller, for emergency admissions. Full regression output is available in eTable 1 in the Supplement.

**Table 2.** Unadjusted and Adjusted Differences in Hospital Selection Between Enrollment Status

Characteristic	Nonemergency				Emergency			
	Hospitalizations, No. (%)		Adjusted Difference (95% CI), %^a^	*P* Value	Hospitalizations, No. (%)		Adjusted Difference (95% CI), %^a^	*P* Value
	TM	MA			TM	MA		
30-d readmissions								
Lowest quintile	595 117 (25.7)	228 274 (23.7)	−1.9 (−2.2 to −1.5)	<.001	1 263 976 (22.2)	490 604 (22.8)	0.0 (0.0 to 0.2)	.60
Quintiles 2-4	1 159 142 (50.1)	522 258 (54.2)	5.1 (4.6 to 5.6)	<.001	2 896 496 (50.9)	1 039 748 (48.3)	−0.1 (−0.3 to 0.0)	.22
Highest quintile	558 439 (24.2)	212 372 (22.1)	−3.2 (−3.5 to −2.9)	<.001	1 530 188 (26.9)	623 335 (28.9)	0.0 (0.0 to 0.2)	.32
Star rating								
1-2	639 283 (26.9)	250 783 (25.9)	−2.6 (−2.9 to −2.2)	<.001	1 826 657 (31.3)	739 628 (34.2)	−0.2 (−0.4 to 0.0)	.03
3	990 219 (41.7)	454 209 (46.9)	5.5 (4.9 to 5.9)	<.001	2 398 139 (41.0)	873 157 (40.4)	0.3 (0.0 to 0.5)	.02
4-5	668 718 (28.2)	235 724 (24.3)	−2.8 (−3.2 to −2.5)	<.001	1 455 502 (24.9)	510 110 (23.6)	0.0 (−0.2 to 0.1)	.65
30-d acute myocardial infarction mortality								
Lowest quintile	639 880 (44.7)	218 578 (42.9)	−1.1 (−1.3 to −0.9)	<.001	1 467 033 (42.2)	515 293 (39.7)	−0.8 (−0.9 to −0.7)	<.001
Quintiles 2-4	416 473 (29.1)	145 473 (28.5)	0.3 (0.0 to 0.6)	.02	1 058 714 (30.5)	403 472 (31.1)	0.4 (0.2 to 0.6)	<.001
Highest quintile	375 205 (26.2)	145 660 (28.6)	0.7 (0.5 to 0.1)	<.001	950 331 (27.3)	390 838 (29.3)	0.4 (0.3 to 06)	<.001
30-d stroke mortality								
Lowest quintile	513 453 (35.4)	181 161 (33.9)	−1.7 (−1.9 to −1.4)	<.001	1 409 930 (39.3)	523 621 (38.2)	−0.1 (−0.2 to 0.0)	.40
Quintiles 2-4	397 952 (27.5)	148 400 (27.8)	1.8 (1.5 to 2.2)	<.001	1 028 907 (28.6)	387 949 (28.3)	0.1 (0.0 to 0.3)	.17
Highest quintile	538 274 (37.1)	204 443 (38.2)	−0.2 (−.04 to 0.1)	.26	1 153 898 (32.1)	460 065 (33.5)	0.0 (−0.2 to 0.0)	.44
30-d coronary artery bypass graft mortality								
Lowest quintile	471 340 (42.7)	153 775 (39.2)	−1.1 (−1.3 to −0.9)	<.001	902 536 (39.3)	306 866 (35.1)	−1.2 (−1.3 to −0.9)	<.001
Quintiles 2-4	369 753 (33.5)	156 630 (39.9)	1.1 (0.8 to 1.4)	<.001	806 390 (35.1)	350 434 (40.1)	0.7 (0.5 to 0.9)	<.001
Highest quintile	263 746 (21.0)	82 399 (20.9)	0.0 (−0.2 to 0.2)	.87	586 931 (25.6)	215 988 (24.7)	0.5 (0.4 to 0.6)	<.001
30-d chronic obstructive pulmonary disease mortality								
Lowest quintile	533 412 (36.6)	176 167 (44.7)	−1.4 (−1.6 to −1.2)	<.001	1 387 711 (38.5)	496 181 (37.7)	−0.4 (−0.6 to −0.2)	<.001
Quintiles 2-4	462 935 (31.8)	174 332 (34.3)	0.0 (−0.3 to 0.3)	.93	1 142 315 (31.7)	443 875 (33.7)	0.4 (0.2 to 0.6)	<.001
Highest quintile	461 314 (31.7)	157 685 (31.0)	1.4 (1.1 to 1.6)	<.001	1 076 513 (29.9)	375 797 (28.6)	0.0 (−0.2 to 0.1)	.63
30-d heart disease mortality								
Lowest quintile	668 376 (45.1)	234 036 (44.4)	−2.0 (−2.3 to −1.8)	<.001	1 655 403 (45.2)	598 222 (43.6)	−1.0 (−1.1 to −0.8)	<.001
Quintiles 2-4	406 367 (27.4)	156 601 (29.7)	2.0 (1.6 to 2.3)	<.001	1 081 716 (29.3)	437 341 (31.9)	0.8 (0.6 to 1.0)	<.001
Highest quintile	407 246 (27.5)	136 381 (25.9)	0.0 (−0.1 to 0.2)	.61	941 702 (25.5)	335 853 (24.5)	0.2 (0.0 to 0.3)	.02

Abbreviations: MA, Medicare Advantage; TM, traditional Medicare.

^a^ Adjusted by a multinomial logit Mundlak model controlling for age, sex, race, zip code mean characteristics, dual-enrollment status, and a flag for enrollment category. Errors are clustered by zip code. The adjusted outcome for each model is estimated using a single multinomial logit model and calculated marginal effects.

In Table 3, we present results from our analysis of plan characteristics associated with hospital quality. The probabilities of being admitted to a low-star hospital for enrollees in plans rated 3 to 3.5, 4 to 4.5, and 5 stars were 4.8 (95% CI, 0.0-9.3), 5.1 (95% CI, 0.0-9.6), and 22.8 (95% CI, 15.0-30.7) percentage points lower, respectively, than those enrolled in plans with 2 to 2.5 stars. Enrollees in contracts owned by national companies and non-HMOs also had lower probabilities of admission to lower-rated hospitals. Contract enrollment, age, and premium were all not statistically significantly associated with admission to a hospital with a high or low star rating. In eTable 2 in the Supplement, we also present results from the multinomial logit models, which yielded similar results.

**Table 3.** Plan Characteristics Associated With Rating of Selected Hospitals^a^

Characteristic	Low-Star Hospital		High-Star Hospital		Low-Readmissions Hospital		High-Readmissions Hospital	
	Difference (95% CI), %	*P* Value	Difference (95% CI), %	*P* Value	Difference (95% CI), %	*P* Value	Difference (95% CI), %	*P* Value
Medicare Advantage star rating								
2-2.5	[Reference]	NA	[Reference]	NA	[Reference]	NA	[Reference]	NA
3-3.5	−4.8 (−9.3 to 0.0)	.04	3.3 (0.6 to 6.0)	.02	3.3 (1.1 to 5.5)	<.001	−4.1 (−8.9 to 0.6)	.09
4-4.5	−5.1 (−9.6 to 0.0)	.03	3.7 (0.8 to 6.5)	.008	3.9 (1.5 to 6.4)	<.001	−3.8 (−8.6 to 1.0)	.13
5	−22.8 (−30.7 to −15.0)	<.001	−3.6 (−9.6 to 2.5)	.28	−2.9 (−8.0 to 2.3)	.32	−17.7 (−25.8 to −9.6)	<.001
Parent company is national	−1.6 (−3.2 to 0.0)	.049	0.3 (−1.4 to 1.9)	.63	0.2 (1.2 to 1.7)	.62	−0.7 (−2.0 to 0.6)	.30
Plan type								
Health maintenance organization	[Reference]	NA	[Reference]	NA	[Reference]	NA	[Reference]	NA
Preferred provider organization	− 2.1 (−3.3 to −0.1)	.005	0.7 (−0.4 to 1.8)	.40	−0.5 (−1.7 to 0.7)	.17	−0.7 (−1.5 to 0.2)	.14
Private fee for service, cost, or other	−2.7 (−4.4 to −0.1)	.007	−0.6 (−2.2 to 0.9)	.23	−2.1 (−4.0 to −0.2)	.01	−1.6 (−2.8 to −0.1)	.04
Contract penetration in county	−2.0 (−3.0 to −0.1)	<.001	−1.0 (−2.0 to 0.0)	.44	−1.2 (−3.0 to 1.0)	.19	−1.3 (−2.0 to 0.0)	.004
Plan premium tertile								
$0	[Reference]	NA	[Reference]	NA	[Reference]	NA	[Reference]	NA
>$0-$50/mo premium	−0.06 (−0.8 to 0.7)	.87	−0.009 (−0.8 to 0.6)	.79	−0.3 (−1.2 to 0.5)	.43	0.4 (−0.2 to 1.1)	.22
>$50/mo premium	−0.7 (−1.6 to 0.09)	.08	1.0 (0.1 to 1.9)	.03	0.7 (−0.3 to 1.6)	.18	0.2 (−0.7 to 1.1)	.63
Contract enrollment tertile								
Tertile 1, <4000	[Reference]	NA	[Reference]	NA	[Reference]	NA	[Reference]	NA
Tertile 2, 4000-24 000	−4.2 (−10.6 to 2.2)	.06	2.5 (−10.6 to 2.2)	.98	1.0 (−1.4 to 3.5)	.21	−1.6 (−4.2 to 1.1)	.15
Tertile 3, >24 000	−3.1 (−9.5 to 3.3)	.05	4.1 (−9.5 to 3.3)	.30	3.0 (0.6 to 5.5)	.02	−0.6 (−3.2 to 2.0)	.23
Contract start year								
<2006	[Reference]	NA	[Reference]	NA	[Reference]	NA	[Reference]	NA
2006-2013	−0.1 (−0.6 to 2.4)	.25	−0.5 (−0.6 to 2.4)	.29	0.8 (−1.8 to 0.3)	.08	0.2 (−0.6 to 1.1)	.29
2014-2019	1.2 (−1.1 to 3.6)	.81	0.8 (−1.1 to 3.6)	.22	2.0 (−2.4 to 6.4)	.47	1.4 (−0.2 to 3.1)	.74

Abbreviation: NA, not applicable.

^a^ Each column is from a separate linear probability model adjusting for the variables in the table and zip code fixed effects among MA enrollees included in the study. Standard errors are clustered on the enrollees contract. Star rating, parent company, contract penetration, contract enrollment, and start year are all at the contract level. Premium and plan type are assessed at the plan level. The contract penetration in county is the association in the probability of admission with a 1% increase in penetration.

In eFigure 1 in the Supplement, we present the unadjusted percentages of MA and TM enrollees who were admitted to hospitals rated 4 stars or higher (as defined in 2016) from 2012 to 2016, stratified by emergency admission status. Over time, there appears to be a persistent trend that MA enrollees had lower rates of unadjusted entry to highly rated hospitals for both emergency admissions (in quarter 1 2012, 25% of MA enrollees and 26% of TM enrollees were admitted to high-quality hospitals; in quarter 4 2016, 25% of MA enrollees and 26% of TM enrollees were admitted to high-quality hospitals) and nonemergency admissions (in quarter 1 2012, 25% of MA enrollees and 29% of TM enrollees were admitted to high-quality hospitals; in quarter 4 2016, 26% of MA enrollees and 31% of TM enrollees were admitted to high-quality hospitals). These trends did not appear to change in 2016 when the star ratings were introduced.

In eTable 3 and eTable 4 in the Supplement, we present the results stratified by different patient subsamples. Admission trends were largely similar across patient subsamples. Notably, while differences were attenuated in rural settings, they were similar between dual-eligible and non–dual eligible enrollees and across plan star ratings. The differences were also similar when further excluding enrollees with prior hospitalizations and only including each enrollee’s first hospitalization of the year. In eTable 5 in the Supplement, we present our alternative model specification with linear probability models including zip code fixed effects, which yielded similar results to our primary models.

## Discussion

We found that after adjusting for patient characteristics and geography, MA enrollees were systematically admitted to average-quality hospitals and less likely to be admitted to hospitals with high star ratings or to those with the lowest star ratings. We observed a similar trend for publicly reported outcome measures for 30-day readmissions and 5 different 30-day mortality measures. We cannot determine whether these differences are due to differences in networks that enrollees were actually able to use or differences in preference; however, the trends were consistent across quality outcomes.

Among enrollees in MA, plan quality had the strongest association with hospital quality. Enrollees in high-rated plans were much less likely to be admitted to lower-quality hospitals compared with enrollees in low-rated plans. We also did not find a consistent association between plan premiums and the quality of networks, indicating that enrollees may not be paying higher premiums in return for higher-quality networks.

Enrollment in higher-rated MA plans, plans that have a national presence, plans with high concentrations in a local market, and non-HMO plans all had lower probabilities of admission to lower-rated hospitals and higher probabilities of admission to higher-rated hospitals. National plans and those with a more robust local presence may be able to more effectively negotiate with hospitals to get better payment arrangements, allowing them to offer higher-quality hospitals in their networks. It is unsurprising that HMOs appeared to be associated with lower-quality admissions, as narrow networks are often a key feature used by HMOs to control costs. As the market power of MA plans increases, they may be able to more effectively negotiate their preferred prices and have a greater ability to include higher-quality hospitals in their networks. Future work that tests the association between market power and admitted hospitals may offer valuable further insight.

There are several possibilities that may have contributed to our findings. Medicare Advantage plans may restrict the hospitals available to patients in their networks,^9^ leading to both the highest- and lowest-quality hospitals being less likely to be included in networks. A similar trend has been found for skilled nursing facilities.^3^ If higher-quality hospitals demand higher reimbursement rates, MA plans may avoid contracting with such hospitals, thereby excluding them from the network. On the other hand, if lower-quality hospitals have higher rehospitalization rates, they will necessarily be costlier to MA plans. Traditional Medicare enrollees living in the same neighborhoods do not face such restrictions on their choice of hospital, which may lead to their use of both higher- and lower-quality hospitals. It is unsurprising that the association was stronger for nonemergency admissions, as planned hospitalizations for surgical procedures and other treatments may be more subject to network design. In the case of emergency hospitalizations, enrollees may be admitted to the nearest hospital; however, it is noteworthy that some differences persisted in selected quality measures. A previous study^20^ found generally broad MA networks in primary care settings; however, hospital care is a more expensive form of health care utilization and may be subject to greater restrictions.

It is notable that all the differences in hospital selection persisted when accounting for zip code in the model. We compared MA and TM enrollees who lived in the same neighborhood directly against each other in our estimates. As such, we do not believe the differences in admitted hospital quality can be explained by differential geographical proximity alone. We cannot rule out the possibility that enrollees in MA and TM have different preferences in the types of hospitals they select, which may explain some of the differences. Medicare Advantage enrollees may prefer to enter average-rated hospitals over high- and low-rated hospitals if these hospitals are different in ways we cannot measure in this study.

There did not appear to be a substantial difference in the use of higher-quality hospitals between TM and MA enrollees in rural areas. These findings may be due to there being fewer hospitals available in rural areas, limiting the ability of MA plans to selectively contract with hospitals and to steer their enrollees to network hospitals.

While this study did not assess the health outcomes that beneficiaries experience, the limited selection of hospitals may lead to poorer health for patients if those patients admitted to average-quality hospitals have worse outcomes than those admitted to the highest-quality hospitals. At the same time, it may be beneficial to enrollees who are steered away from the lowest-quality hospitals. To address this concern, MA networks could be incorporated into annual calculation of MA star ratings and reported on each year. Currently, when enrollees choose MA plans, there is limited information about the breadth and quality of hospitals available in the plans’ networks that can be used when making enrollment decisions. The Centers for Medicare and Medicaid Services could take steps to make this more transparent.

### Limitations

This study has several limitations. Hospital star ratings are new and not necessarily a validated measure of quality. Early work has found that higher star ratings may be associated with higher quality of care for some conditions, but other work has found teaching hospitals and hospitals that treat low-income patients may be penalized.^21,22^ Despite these limitations, the star ratings are calculated on the basis of a variety of quality measures that may be important to patient outcomes, and the differences in admitted hospital quality persisted for mortality and readmissions rates, indicating that the differences in the rates of admission we detected may not be due to the idiosyncrasies of the star rating system alone. While this study included Medicare beneficiaries both older and younger than 65 years, the publicly reported measures we use are generally calculated only from those older than 65 years. As such, these quality measures may be less sensitive to the experience of younger beneficiaries. Another key limitation of this study is that we must infer from MA plan member health care utilization patterns to discern which hospitals are in a given MA plan’s network, as accurate official hospital networks are not publicly available. Furthermore, while we adjusted for sociodemographic characteristics and comorbidities, it is possible that unobserved factors associated with both MA enrollment and hospital choice may have influenced these results. Nonetheless, the fact that we observed large differences in quality strongly suggests that MA plans restrict hospitals from membership in their networks. We are also limited in that we only have data on MA hospitalizations from hospitals that receive additional funding from Medicaid Disproportionate Share Hospital payments or medical education. While this covers most MA hospitalizations in the country, it may underrepresent MA enrollees living in rural areas.

## Conclusions

With its continued growth, MA has become a vital component of the health care system. This study found differences in the quality of hospitals admitting MA patients compared with TM patients, suggesting that policy makers should monitor the quality of hospitals available in MA plans’ network and make this information available to enrollees.

## Notes

Supplement. **eTable 1.** Full Regression Output for Primary Models **eFigure 1.** Trends in Share of MA and TM Patients Admitted to 4-5 Star Hospitals Between 2012-2016 **eFigure 2.** Share of MA and TM Patients Admitted to Hospitals in Neighborhoods With Different Proximity to Hospitals, Emergency and Non-emergency Admissions **eTable 2.** Multinomial Logit Regression Comparison **eTable 3.** Alternative Model and Sample Specifications: Star Rating **eTable 4.** Alternative Model and Sample Specifications: Readmissions **eTable 5.** Comparing Primary Outcomes Using Linear Probability Models Adjusted for Zipcode Fixed Effects
