# Supplementary material for: Comparison of the Quality of Hospitals That Admit Medicare Advantage Patients vs Traditional Medicare Patients
Source: JAMA Netw Open. 2020 Jan 15;3(1):e1919310. doi: 10.1001/jamanetworkopen.2019.19310 (PMC6991262; doi:10.1001/jamanetworkopen.2019.19310)
Supplement: Supplement. — eTable 1. Full Regression Output for Primary Models eFigure 1. Trends in Share of MA and TM Patients Admitted to 4-5 Star Hospitals Between 2012-2016 eFigure 2. Share of MA and TM Patients Admitted to Hospitals in Neighborhoods With Different Proximity to Hospitals, Emergency and Non-emergency Admissions eTable 2. Multinomial Logit Regression Comparison eTable 3. Alternative Model and Sample Specifications: Star Rating eTable 4. Alternative Model and Sample Specifications: Readmissions eTable 5. Comparing Primary Outcomes Using Linear Probability Models Adjusted for Zipcode Fixed Effects [file jamanetwopen-3-e1919310-s001.pdf]

Supplementary Online Content

Meyers DJ, Trivedi AN, Mor V, Rahman M. Comparison of the quality of hospitals that admit Medicare Advantage patients vs traditional Medicare patients. *JAMA Netw Open*. 2020;3(1):e1919310. doi:10.1001/jamanetworkopen.2019.19310

eTable 1. Full Regression Output for Primary Models

eFigure 1. Trends in Share of MA and TM Patients Admitted to 4-5 Star Hospitals Between 2012-2016

eFigure 2. Share of MA and TM Patients Admitted to Hospitals in Neighborhoods With Different Proximity to Hospitals, Emergency and Non-emergency Admissions

eTable 2. Multinomial Logit Regression Comparison

eTable 3. Alternative Model and Sample Specifications: Star Rating

eTable 4. Alternative Model and Sample Specifications: Readmissions

eTable 5. Comparing Primary Outcomes Using Linear Probability Models Adjusted for Zipcode Fixed Effects

This supplementary material has been provided by the authors to give readers additional information about their work.

© 2020 Meyers DJ et al. *JAMA Network Open.*

eTable 1: Full regression output for primary models

	Star Rating				30-Day Readmissions			
	Non-Emergency		Emergency		Non-Emergency		Emergency	
	Low Star (1)	High Star (5)	Low Star (1)	High Star (5)	Lowest Quintile	Highest Quintile	Lowest Quintile	Highest Quintile
MA	-0.239**	-0.234**	-0.017*	-0.009	-0.195**	-0.273**	0.006	0.008
	(-0.261 - -0.217)	(-0.258 - -0.210)	(-0.030 - -0.004)	(-0.022 - 0.004)	(-0.221 - -0.168)	(-0.296 - -0.250)	(-0.008 - 0.019)	(-0.006 - 0.021)
Age	-0.005**	-0.001**	-0.003**	0.001**	0.002**	-0.007**	0.003**	-0.004**
	(-0.006 - -0.005)	(-0.002 - -0.001)	(-0.003 - -0.003)	(0.001 - 0.001)	(0.001 - 0.002)	(-0.008 - -0.006)	(0.003 - 0.004)	(-0.004 - -0.003)
Race, black	0.076**	-0.086**	0.105**	-0.065**	-0.049**	0.105**	-0.064**	0.129**
	(0.059 - 0.093)	(-0.103 - -0.069)	(0.089 - 0.121)	(-0.081 - -0.049)	(-0.067 - -0.030)	(0.088 - 0.122)	(-0.081 - -0.046)	(0.112 - 0.146)
Race, other	-0.004	0.062**	0.009	0.041**	-0.036**	0.022	-0.071**	0.045**
	(-0.029 - 0.022)	(0.037 - 0.087)	(-0.017 - 0.036)	(0.011 - 0.071)	(-0.062 - -0.010)	(-0.004 - 0.047)	(-0.101 - -0.042)	(0.021 - 0.069)
Race, Hispanic	0.129**	0.096**	0.010	0.080**	0.042	0.212**	0.083**	0.095**
	(0.092 - 0.166)	(0.057 - 0.136)	(-0.016 - 0.035)	(0.048 - 0.112)	(-0.001 - 0.085)	(0.170 - 0.255)	(0.041 - 0.126)	(0.058 - 0.131)
Race, Asian	0.132**	-0.035*	0.045**	-0.034**	-0.054**	0.122**	-0.055**	0.066**
	(0.106 - 0.158)	(-0.063 - -0.007)	(0.027 - 0.064)	(-0.054 - -0.014)	(-0.083 - -0.025)	(0.095 - 0.150)	(-0.077 - -0.033)	(0.047 - 0.085)
Race, AI/NA	0.142*	-0.330**	-0.113*	-0.593**	0.201	-0.410**	0.315**	-0.238**
	(0.012 - 0.272)	(-0.493 - -0.168)	(-0.212 - -0.014)	(-0.768 - -0.418)	(-0.031 - 0.433)	(-0.561 - -0.260)	(0.155 - 0.474)	(-0.378 - -0.097)
Gender	-0.045**	0.005	-0.033**	0.004	0.012**	-0.062**	-0.001	-0.022**
	(-0.053 - -0.038)	(-0.002 - 0.012)	(-0.038 - -0.028)	(-0.001 - 0.009)	(0.005 - 0.019)	(-0.069 - -0.054)	(-0.007 - 0.004)	(-0.027 - -0.016)
Dual Eligibility	0.131**	-0.140**	0.092**	-0.086**	-0.095**	0.109**	-0.059**	0.082**
	(0.118 - 0.144)	(-0.153 - -0.127)	(0.083 - 0.102)	(-0.095 - -0.076)	(-0.109 - -0.081)	(0.095 - 0.123)	(-0.070 - -0.048)	(0.072 - 0.093)
Distance to Nearest High Quality Facility	0.011**	-0.040**	0.004	-0.107**	-0.054**	0.001**	-0.139**	0.002**
	(0.007 - 0.014)	(-0.043 - -0.036)	(-0.001 - 0.009)	(-0.112 - -0.101)	(-0.056 - -0.052)	(0.001 - 0.001)	(-0.142 - -0.135)	(0.001 - 0.002)
Distance to Nearest Average Quality Facility	0.050**	0.030**	0.143**	0.098**	0.003**	-0.055**	0.001**	-0.138**
	(0.047 - 0.053)	(0.028 - 0.032)	(0.138 - 0.148)	(0.094 - 0.103)	(0.002 - 0.003)	(-0.056 - -0.053)	(0.001 - 0.002)	(-0.142 - -0.134)
Distance to Average Low Quality Facility	-0.048**	-0.000	-0.129**	-0.001	0.054**	0.048**	0.151**	0.153**
	(-0.050 - -0.046)	(-0.001 - 0.001)	(-0.133 - -0.125)	(-0.002 - 0.000)	(0.051 - 0.057)	(0.045 - 0.051)	(0.147 - 0.155)	(0.148 - 0.158)
Constant	-0.318	-3.065**	-1.156	-5.711**	3.217**	-1.844**	1.148	-2.117**
	(-1.257 - 0.621)	(-3.918 - -2.212)	(-2.367 - 0.055)	(-6.995 - -4.427)	(2.348 - 4.085)	(-2.760 - -0.927)	(-0.012 - 2.307)	(-3.286 - -0.948)
Observations	3,187,171	3,187,171	7,661,256	7,661,256	3,187,171	3,187,171	7,661,256	7,661,256

*All models are multinomial logit Mundlak models.

© 2020 Meyers DJ et al. *JAMA Network Open.*

eFigure 1: Trends in share of MA and TM patients admitted to 4-5 star hospitals between 2012-2016

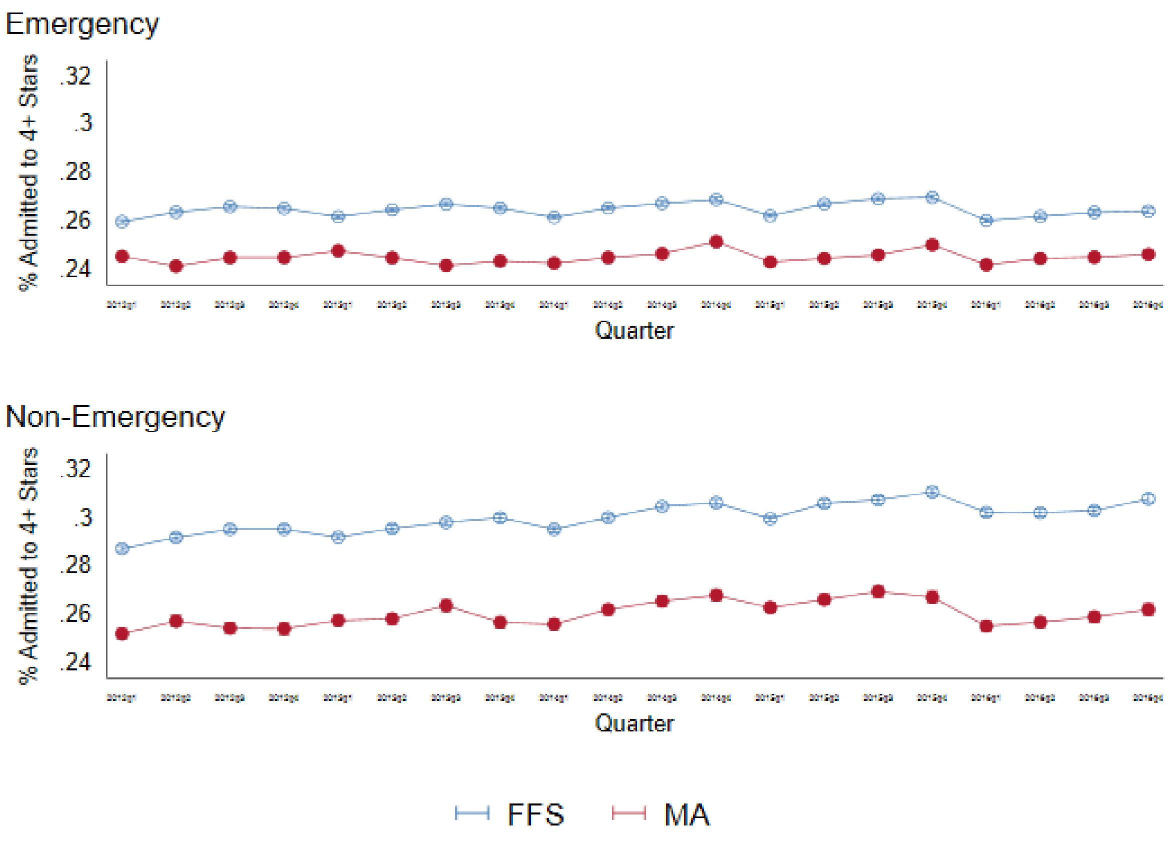

Notes: Figure shows the unadjusted admission rates into a 4+ star hospital by FFS and MA status at the time of admission quarterly from 2012-2016. Panel A displays admissions for emergency admissions while Panel B displays non-emergency admissions. Star rating of hospital is defined in 2016 and applied backwards to previous years.

© 2020 Meyers DJ et al. *JAMA Network Open.*

eFigure 2: Share of MA and TM patients admitted to hospitals in neighborhoods with different proximity to hospitals, emergency and non-emergency admissions

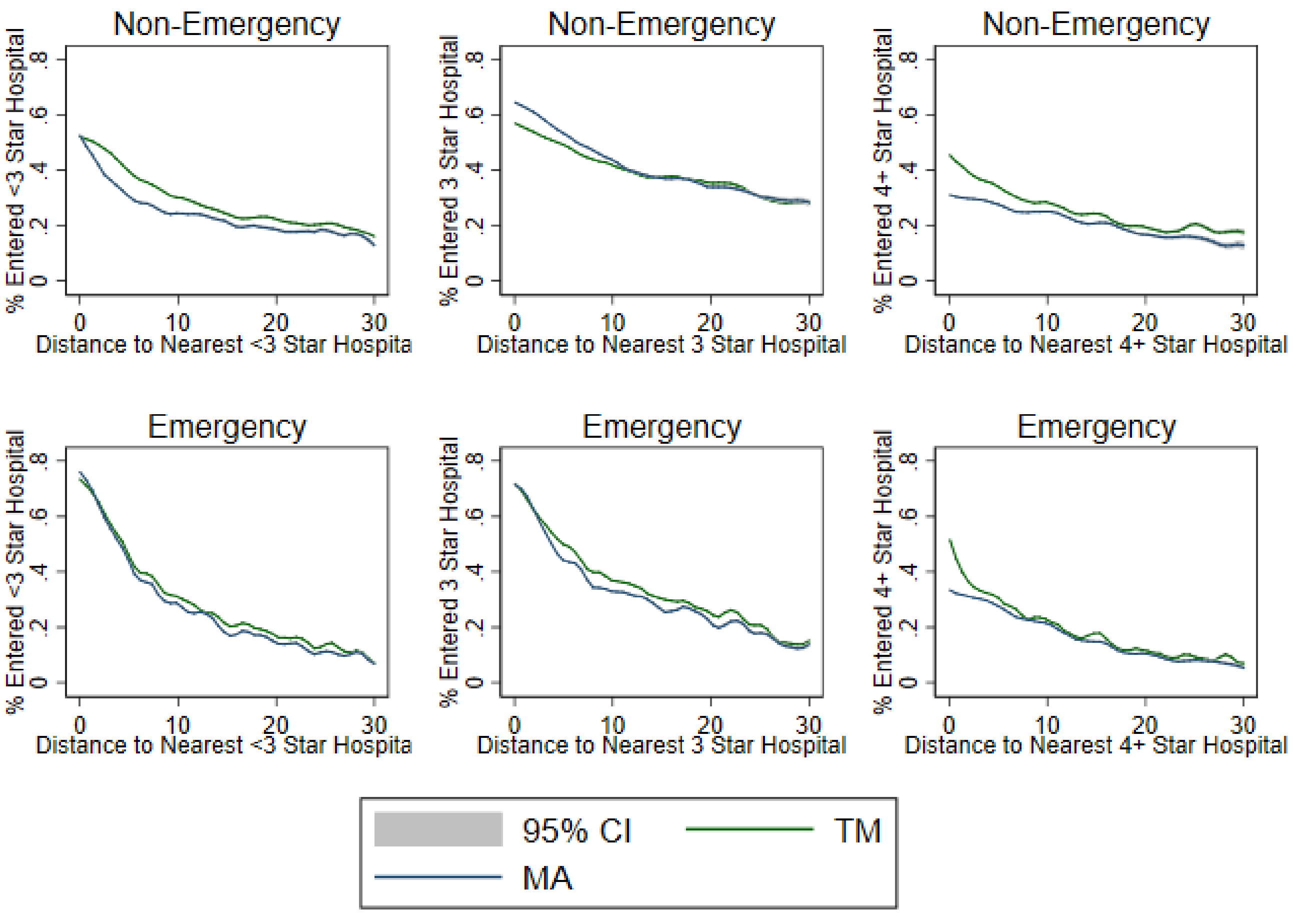

© 2020 Meyers DJ et al. *JAMA Network Open.*

eTable 2: Multinomial Logit Regression Comparison

		Non-Emergency				Emergency			
		TM n (%)	MA n (%)	Adjusted Difference	95% CI	TM n (%)	MA n (%)	Adjusted Difference	95% CI
Star Rating	1-2 Stars	639,283 (26.9)	250,783 (25.9)	-2.8%**	(-2.9, -2.7)	1,826,657 (31.3)	739,628 (34.2)	0.4%**	(0.4, 0.5)
	3 Stars	990,219 (41.7)	454,209 (46.9)	5.4%**	(5.3, 5.7)	2,398,139 (41.0)	873,157 (40.4)	-0.4%**	(-0.5, -0.3)
	4-5 Stars	668,718 (28.2)	235,724 (24.3)	-2.6%**	(-2.7, -2.5)	1,455,502 (24.9)	510,110 (23.6)	0.0%	(-0.0, -0.0)
30-Day Readmissions	Lowest Quintile	595,117 (25.7)	228,274 (23.7)	-1.8**	(-1.9, -1.8)	1,263,976 (22.2)	490,604 (22.8)	0.5**	(0.4, 0.5)
	Quintiles 2-4	1,159,142 (50.1)	522,258 (54.2)	5.6**	(5.5, 5.8)	2,896,496 (50.9)	1,039,748 (48.3)	-0.9**	(-0.9, -0.8)
	Highest Quintile	558,439 (24.2)	212,372 (22.1)	-3.8**	(-3.9, -3.7)	1,530,188 (26.9)	623,335 (28.94)	0.4**	(0.4, 0.5)
30-Day AMI Mortality	Lowest Quintile	639,880 (44.7)	218,578 (42.9)	-2.5**	(-2.5, -2.4)	1,467,033 (42.2)	515,293 (39.7)	-1.7**	(-1.8, -1.7)
	Quintiles 2-4	416,473 (29.1)	145,473 (28.5)	1.7**	(1.6, 1.8)	1,058,714 (30.5)	403,472 (31.1)	1.0**	(0.9, 1.1)
	Highest Quintile	375,205 (26.2)	145,660 (28.6)	0.7**	(0.7, 0.8)	950,331 (27.3)	390,838 (29.3)	0.8**	(0.7, 0.8)
30-Day Stroke Mortality	Lowest Quintile	513,453 (35.4)	181,161 (33.9)	-2.8**	(-2.7, -2.5)	1,409,930 (39.3)	523,621 (38.2)	-0.9**	(-1.0, -0.9)
	Quintiles 2-4	397,952 (27.5)	148,400 (27.8)	-2.3**	(2.2, 2.4)	1,028,907 (28.6)	387,949 (28.3)	0.2**	(0.1, 0.3)
	Highest Quintile	538,274 (37.1)	204,443 (38.2)	0.3**	(0.2, 0.4)	1,153,898 (32.1)	460,065 (33.5)	0.7**	(0.7, 0.8)
30-Day CABG Mortality	Lowest Quintile	471,340 (42.7)	153,775 (39.2)	-1.9**	(-2.1, -1.8)	902,536 (39.3)	306,866 (35.1)	-1.8**	(-1.8, -1.7)
	Quintiles 2-4	369,753 (33.5)	156,630 (39.9)	2.2**	(2.1, 2.3)	806,390 (35.1)	350,434 (40.1)	1.1**	(1.1, 1.2)
	Highest Quintile	263,746 (20.98)	82,399 (20.9)	-0.2**	(-0.3, -0.1)	586,931 (25.6)	215,988 (24.7)	0.6**	(0.6, 0.7)
30-Day COPD Mortality	Lowest Quintile	533,412 (36.6)	176,167 (44.7)	-2.7**	(-2.7, 2.6)	1,387,711 (38.5)	496,181 (37.7)	-1.5**	(-1.6, -1.4)
	Quintiles 2-4	462,935 (31.8)	174,332 (34.3)	2.8**	(2.6, 2.9)	1,142,315 (31.7)	443,875 (33.7)	2.0**	(1.9, 2.1)
	Highest Quintile	461,314 (31.7)	157,685 (31.0)	-0.0	(0.0, 0.0)	1,076,513 (29.9)	375,797 (28.6)	-0.5**	(-0.5, -0.4)
30-Day Heart Disease Mortality	Lowest Quintile	668,376 (45.1)	234,036 (44.4)	-4.1**	(-4.1, -4.0)	1,655,403 (45.2)	598,222 (43.6)	-2.7**	(-2.8, -2.7)
	Quintiles 2-4	406,367 (27.4)	156,601 (29.7)	4.2**	(4.1, 4.3)	1,081,716 (29.3)	437,341 (31.9)	2.5**	(2.4, 2.6)
	Highest Quintile	407,246 (27.5)	136,381 (25.9)	-0.1*	(-0.2, -0.0)	941,702 (25.5)	335,853 (24.5)	0.2**	(0.2, 0.3)

* denotes p<0.05, ** denotes p<0.001

Results in the multinomial logit model come from a single multinomial in each emergency category, adjusting for gender, age, dual eligibility status, and race/ethnicity.

© 2020 Meyers DJ et al. *JAMA Network Open.*

eTable 3: Alternative model and sample specifications: star rating

		Emergency		Non-Emergency	
		Adjusted Difference		Adjusted Difference	
		(95% CI)		(95% CI)	
Dual Eligible	Low Rated (1-2 Stars)	-1.3%**	(-1.3--1.1)	-0.3%**	(-0.3--0.1)
	Average Rated (3 Stars)	6.3%**	(6-6.5)	0.00%	(0-0.1)
	High Rated (4-5 Stars)	-2.1%**	(-2.3--1.9)	0.10%	(0-0.1)
Not Dual Eligible	Low Rated (1-2 Stars)	-3.4%**	(-3.5--3.1)	0.00%	(-0.1-0)
	Average Rated (3 Stars)	3.2%**	(3-3.2)	0.4%**	(0.3-0.4)
	High Rated (4-5 Stars)	-2.0%**	(-2.1--1.8)	-0.1%**	(-0.1-0)
Rural	Low Rated (1-2 Stars)	-0.10%	(-0.2-0.1)	-0.10%	(-0.2-0)
	Average Rated (3 Stars)	0.10%	(-0.1-0.3)	-0.10%	(-0.2-0.1)
	High Rated (4-5 Stars)	-0.10%	(-0.3-0)	0.2%**	(0-0.3)
Not Rural	Low Rated (1-2 Stars)	-2.1%**	(-2.1--1.9)	-0.2%**	(-0.2--0.1)
	Average Rated (3 Stars)	4.8%**	(4.6-4.9)	0.4%**	(0.3-0.4)
	High Rated (4-5 Stars)	-2.6%**	(-2.7--2.4)	-0.2%**	(-0.2-0)
3+ Comorbidities	Low Rated (1-2 Stars)	-1.8%**	(-1.9--1.7)	-0.1%**	(-0.1-0)
	Average Rated (3 Stars)	4.4%**	(4.2-4.5)	0.3%**	(0.2-0.3)
	High Rated (4-5 Stars)	-2.2%**	(-2.3--2)	-0.1%**	(-0.1-0)
Diagnosis 1	Low Rated (1-2 Stars)	-1.1%**	(-1.4--0.8)	-0.9%**	(-1.2--0.6)
	Average Rated (3 Stars)	1.8%**	(1.4-2.2)	2.5%**	(1.9-2.9)
	High Rated (4-5 Stars)	-2.3%**	(-2.6--1.9)	-3.0%**	(-3.4--2.5)
Diagnosis 2	Low Rated (1-2 Stars)	3.3%**	(-1.9--0.4)	-0.10%	(-0.3-0)
	Average Rated (3 Stars)	3.3%**	(2.3-4.1)	0.2%**	(0-0.3)
	High Rated (4-5 Stars)	-1.9%**	(-2.7--1.1)	0.00%	(-0.1-0.1)
Diagnosis 3	Low Rated (1-2 Stars)	-0.10%	(-0.3-0.1)	-0.6%**	(-0.8--0.3)
	Average Rated (3 Stars)	0.5%**	(0.1-0.8)	0.5%**	(0.2-0.8)
	High Rated (4-5 Stars)	-0.20%	(-0.5-0)	0.10%	(-0.1-0.3)
Adjusting for ICU use and Elixhuaser Index	Low Rated (1-2 Stars)	-1.7%**	(-1.7--1.5)	-0.3%**	(-0.3--0.2)
	Average Rated (3 Stars)	3.8%**	(3.6-3.9)	0.4%**	(0.3-0.4)
	High Rated (4-5 Stars)	-2.1%**	(-2.1--1.9)	-0.1%**	(-0.1-0)
Excluding prior hospitalization and subsequent admissions	Low Rated (1-2 Stars)	-1.7%**	(-1.8--1.5)	-0.2%**	(-0.2--0.1)
	Average Rated (3 Stars)	3.9%**	(3.7-3.9)	0.3%**	(0.2-0.3)
	High Rated (4-5 Stars)	-2.4%**	(-2.4--2.2)	-0.1%**	(-0.1-0)
Propensity Score Weighed	Low Rated (1-2 Stars)	-1.2**	(-1.3--1.1)	-0.0	(-0.0-0.0)
	Average Rated (3 Stars)	3.1**	(3.0-3.3)	0.2**	(0.2-0.3)
	High Rated (4-5 Stars)	-1.8**	(-1.9--1.7)	-0.1**	(-0.2--0.0)

Notes: * denotes p<0.05, ** denotes p<0.001. Dual eligibility status comes from the master beneficiary summary file. Rurality is of the patient's zipcode. 3+ comorbidities are based off of the Elixhuaser comorbidity index of the given

© 2020 Meyers DJ et al. *JAMA Network Open.*

hospitalization's diagnoses codes. The top three diagnoses for non-emergency were hip and knee osteoarthritis, and Coronary atherosclerosis. The top three diagnoses for emergency admissions are septicemia, pneumonia, and acute myocardial infarction. The second to last sensitivity rows additionally include ICU use and the Elixhuaser index in the model as covariates. In the final check, we exclude patients who had a hospitalization or nursing home stay in the prior 6 months, and only include the first hospitalization of the year in the models.

© 2020 Meyers DJ et al. *JAMA Network Open.*

eTable 4: Alternative model and sample specifications: readmissions

		Emergency		Non-Emergency	
		Adjusted Difference	(95% CI)	Adjusted Difference	(95% CI)
Dual Eligible	Quintile 1	-1.1**	(-1.3--1.0)	0.2**	(0.1-0.3)
	Quintiles 2-4	4.5**	(4.2-4.8)	-0.0	(-0.2-0.0)
	Quintile 5	-3.1**	(-3.3--2.9)	-0.0	(-0.2-0.0)
Not Dual Eligible	Quintile 1	-1.5**	(-1.6--1.4)	0.2**	(0.2-0.3)
	Quintiles 2-4	3.3**	(3.2-3.4)	-0.0	(-0.1-0.0)
	Quintile 5	-1.6**	(-1.7--1.5)	-0.1**	(-0.2-0.0)
Rural	Quintile 1	-0.1	(-0.3-0.0)	0.4**	(0.3-0.5)
	Quintiles 2-4	0.3*	(0.0-0.5)	-0.4**	(-0.5--0.2)
	Quintile 5	-0.1	(-0.3-0.0)	-0.0	(0.1-0.0)
Not Rural	Quintile 1	-1.7**	(-1.8--1.6)	0.2**	(0.1-0.2)
	Quintiles 2-4	4.4**	(4.3-4.5)	0.0	(0.0-0.0)
	Quintile 5	-2.4**	(-2.5--2.3)	-0.2**	(-0.2--0.1)
3+ Comorbidities	Quintile 1	-1.3**	(-1.5--1.3)	0.2**	(0.2-0.3)
	Quintiles 2-4	3.8**	(3.7-4.0)	0.0	(0.0-0.0)
	Quintile 5	-2.2**	(-2.3--2.0)	-0.2**	(-0.2--0.1)
Diagnosis 1	Quintile 1	-2.2**	(-2.5--1.8)	0.4**	(0.1-0.5)
	Quintiles 2-4	2.8**	(2.3-3.1)	-0.2	(-0.4-0.0)
	Quintile 5	-0.5**	(-0.8--0.2	-0.1	(-0.3-0.0)
Diagnosis 2	Quintile 1	-3.2**	(-3.8--2.8)	-0.1	(-0.4-0.1)
	Quintiles 2-4	3.9**	(3.4-4.4)	0.3*	(0.0-0.7)
	Quintile 5	-0.7**	(-1.0--0.3)	-0.2	(-0.4-0.0)
Diagnosis 3	Quintile 1	-2.9**	(-3.6--2.3)	0.1	(-0.1-0.3)
	Quintiles 2-4	4.2**	(3.4-5.1)	0.3	(-0.0-0.6)
	Quintile 5	-1.3**	(-1.9--0.5)	-0.3*	(-0.6--0.0)
Adjusting for ICU use and Elixhuaser Index	Quintile 1	-1.4**	(-1.5--1.3)	0.2**	(0.2-0.3)
	Quintiles 2-4	3.6**	(3.4-3.7)	0.0	(-0.0-0.0)
	Quintile 5	-1.9**	(-2.0--1.8)	-0.2**	(-0.2--0.1)
Excluding prior hospitalization and subsequent admissions	Quintile 1	-1.6**	(-1.7--1.5)	0.2**	(0.1-0.2)
	Quintiles 2-4	3.4**	(3.3-3.6)	-0.0	(-0.1-0.0)
	Quintile 5	-1.6**	(-1.7--1.5)	-0.0*	(-0.2--0.0)
Propensity Score Weighed	Quintile 1	-1.1**	(-1.1--1.0)	0.2**	(0.2-0.2)
	Quintiles 2-4	2.8**	(2.7-2.9)	-0.0	(0.0-0.0)
	Quintile 5	-1.5**	(-1.6--1.4)	-0.1**	(-0.2--0.1)

Notes: * denotes p<0.05, ** denotes p<0.001. Dual eligibility status comes from the master beneficiary summary file. Rurality is of the patient's zipcode. 3+ comorbidities are based off of the Elixhuaser comorbidity index of the given

© 2020 Meyers DJ et al. *JAMA Network Open.*

hospitalization's diagnoses codes. The top three diagnoses for non-emergency were hip and knee osteoarthritis, and Coronary atherosclerosis. The top three diagnoses for emergency admissions are septicemia, pneumonia, and acute myocardial infarction. The second to last sensitivity rows additionally include ICU use and the Elixhuaser index in the model as covariates. In the final check, we exclude patients who had a hospitalization or nursing home stay in the prior 6 months, and only include the first hospitalization of the year in the models.

© 2020 Meyers DJ et al. *JAMA Network Open.*

eTable 5: Comparing primary outcomes using linear probability models adjusted for zipcode fixed effects

		Non-Emergency				Emergency			
		TM n (%)	MA n (%)	Adjusted Difference	95% CI	TM n (%)	MA n (%)	Adjusted Difference	95% CI
Star Rating	1-2 Stars	639,283 (26.9)	250,783 (25.9)	-1.7**	(-1.8--1.6)	1,826,657 (31.3)	739,628 (34.2)	-0.2**	(-0.3--0.2)
	3 Stars	990,219 (41.7)	454,209 (46.9)	3.8**	(3.7-4.0)	2,398,139 (41.0)	873,157 (40.4)	0.4**	(0.3-0.4)
	4-5 Stars	668,718 (28.2)	235,724 (24.3)	-2.1**	(-2.2--2.0)	1,455,502 (24.9)	510,110 (23.6)	0.08**	(0.1-0.03)
30-Day Readmissions	Lowest Quintile	595,117 (25.7)	228,274 (23.7)	-1.4**	(-1.4--1.3)	1,263,976 (22.2)	490,604 (22.8)	0.2**	(0.2-0.3)
	Quintiles 2-4	1,159,142 (50.1)	522,258 (54.2)	3.6**	(3.5-3.7)	2,896,496 (50.9)	1,039,748 (48.3)	0	(0.0-0.0)
	Highest Quintile	558,439 (24.2)	212,372 (22.1)	-2.2**	(-2.3--2.1)	1,530,188 (26.9)	623,335 (28.94)	-0.2**	(-0.3--0.2)
30-Day AMI Mortality	Lowest Quintile	639,880 (44.7)	218,578 (42.9)	0.6%**	(0.5-0.6)	1,467,033 (42.2)	515,293 (39.7)	0.3%**	(0.2-0.3)
	Quintiles 2-4	416,473 (29.1)	145,473 (28.5)	-0.8%**	(-0.8--0.6)	1,058,714 (30.5)	403,472 (31.1)	-0.1%**	(-0.1-0)
	Highest Quintile	375,205 (26.2)	145,660 (28.6)	-1.1%**	(-1.1--0.9)	950,331 (27.3)	390,838 (29.3)	-0.9%**	(-0.9--0.8)
30-Day Stroke Mortality	Lowest Quintile	513,453 (35.4)	181,161 (33.9)	-0.2%**	(-0.2-0)	1,409,930 (39.3)	523,621 (38.2)	-0.2%**	(-0.2--0.1)
	Quintiles 2-4	397,952 (27.5)	148,400 (27.8)	0.4%**	(0.3-0.5)	1,028,907 (28.6)	387,949 (28.3)	-0.1%**	(-0.1-0)
	Highest Quintile	538,274 (37.1)	204,443 (38.2)	-1.4%**	(-1.4--1.2)	1,153,898 (32.1)	460,065 (33.5)	-0.1%**	(-0.1-0)
30-Day CABG Mortality	Lowest Quintile	471,340 (42.7)	153,775 (39.2)	-1.4%**	(-1.5--1.3)	902,536 (39.3)	306,866 (35.1)	-1.1%**	(-1.1--0.9)
	Quintiles 2-4	369,753 (33.5)	156,630 (39.9)	0.00%	(0-0.1)	806,390 (35.1)	350,434 (40.1)	-0.10%	(-0.1-0)
	Highest Quintile	263,746 (20.98)	82,399 (20.9)	0.00%	(-0.1-0)	586,931 (25.6)	215,988 (24.7)	0.3%**	(0.2-0.3)
30-Day COPD Mortality	Lowest Quintile	533,412 (36.6)	176,167 (44.7)	-1.2%**	(-1.3--1.1)	1,387,711 (38.5)	496,181 (37.7)	-0.6%**	(-0.6--0.5)
	Quintiles 2-4	462,935 (31.8)	174,332 (34.3)	-0.9%**	(-1--0.8)	1,142,315 (31.7)	443,875 (33.7)	-0.6%**	(-0.6--0.5)
	Highest Quintile	461,314 (31.7)	157,685 (31.0)	1.3%**	(1.2-1.4)	1,076,513 (29.9)	375,797 (28.6)	0.2%**	(0.1-0.2)
30-Day Heart Disease Mortality	Lowest Quintile	668,376 (45.1)	234,036 (44.4)	-1.8%**	(-1.8--1.7)	1,655,403 (45.2)	598,222 (43.6)	-1.1%**	(-1.1--1)
	Quintiles 2-4	406,367 (27.4)	156,601 (29.7)	0.6%**	(0.5-0.7)	1,081,716 (29.3)	437,341 (31.9)	0.4%**	(0.3-0.4)
	Highest Quintile	407,246 (27.5)	136,381 (25.9)	0.0%**	(0-0)	941,702 (25.5)	335,853 (24.5)	0.3%**	(0.2-0.3)

© 2020 Meyers DJ et al. *JAMA Network Open.*
